# A negative nontreponemal and/or specific antitreponemal IgM test does not exclude active infectious syphilis: evidence from a rabbit infectivity test: A case report

**DOI:** 10.1097/MD.0000000000004520

**Published:** 2016-08-07

**Authors:** Li-Rong Lin, Man-Li Tong, Kun Gao, Xiao-Zhen Zhu, Jin-Yi Fan, Wei-Hong Zheng, Shu-Lian Li, Hui-Ling Lin, Li-Li Liu, Tian-Ci Yang

**Affiliations:** a Zhongshan Hospital, Medical College of Xiamen University; b Xiamen Huli District Maternity and Child Care Hospital, Xiamen, China.

**Keywords:** active infectious syphilis, antitreponemal IgM test, nontreponemal test, rabbit infectivity test

## Abstract

**Background:**

The diagnostic criteria for active infectious syphilis in the clinic are important matter of controversy and debate. So far, clinicians habitually do use the negative results of the nontreponemal and/or the specific antitreponemal IgM as the evidences of disease-free or active infection-free status.

**Method:**

We present a case study involving a patient who was admitted to Zhongshan Hospital because of cerebral infarct. Clinical examination indicated he had a history of latent syphilis with negative nontreponemal and specific antitreponemal IgM tests. The cerebrospinal fluid sample from the patient was inoculated into seronegative New Zealand rabbit.

**Results:**

Motile *Treponema pallidum* was detected by a rabbit infectivity test in the patient's cerebrospinal fluid. This syphilis strain was confirmed by DNA subtyping form of “centers for disease control subtype/tp0548 sequence type”, and the strain type was 14d/f. Treatment with benzathine penicillin provided no apparent benefit, but treatment with aqueous crystalline penicillin G, especially recommended for neurosyphilis, led to disease regression. No evidence of cerebral infarct was observed during a 2-year follow-up period.

**Conclusion:**

The definitive differential diagnosis of active infectious syphilis should be reconsidered. Moreover, selecting the appropriate penicillin preparation is important because *T pallidum* can reside in sequestered sites. It is necessary to treat a patient with known invasion of the central nervous system with aqueous crystalline penicillin G, if previous treatment for syphilis failed and patients had some clinical neurological presentation that is otherwise unexplained, but that could represent neurosyphilis. Additional studies are needed to confirm the results in other syphilis patients.

## 1 Introduction

Currently, there are still controversies existing on which criteria to be used to diagnose cases of active infectious syphilis in a clinical setting.^[1]^ Clinicians generally rely on serological markers to diagnose active syphilis. For example, syphilis-specific IgM cn be detected as early as 2 weeks after infection,^[2]^ nontreponemal antibodies can be detected 5 to 7 weeks after initial infection, and both of these are generally correlated with disease activity.^[3]^ The rabbit infectivity test (RIT) is another method used to establish infection with viable *Treponema pallidum*, and it has historically been considered as the gold standard for detecting active syphilis. This method has a test sensitivity high enough to detect as few as a single organism when repeated passages are performed in rabbits.^[4]^ However, to perform this test, access to an animal facility is required, and it is extremely time-consuming and expensive.^[5]^ For this reason, it is most commonly used in a research setting, but is impractical and expensive as a routine diagnostic procedure.

Without treatment, an active syphilis infection can develop into symptomatic late syphilis. The most dreaded complications of this disease are neurosyphilis and the involvement of the aortic valve and root.^[1]^ Here, we report a patient with cerebral infarct and presumed latent syphilis with negative serum rapid plasma reagin (RPR) and syphilis-specific IgM tests, even though motile *T pallidum* was detected in the patient's cerebrospinal fluid (CSF) by RIT. This study was approved by the Institutional Ethics Committee of Zhongshan Hospital, and it was performed in compliance with national legislation and the guidelines of the Declaration of Helsinki. Written patient consent was obtained according to institutional guidelines. All rabbit experiments were performed using protocols approved by the animal experimental ethics committee of the Medical College of Xiamen University. The CSF sample (undiluted) was inoculated into seronegative New Zealand white male mature rabbits as previously reported.^[6]^

## 2 Case report

**Figure 1:**
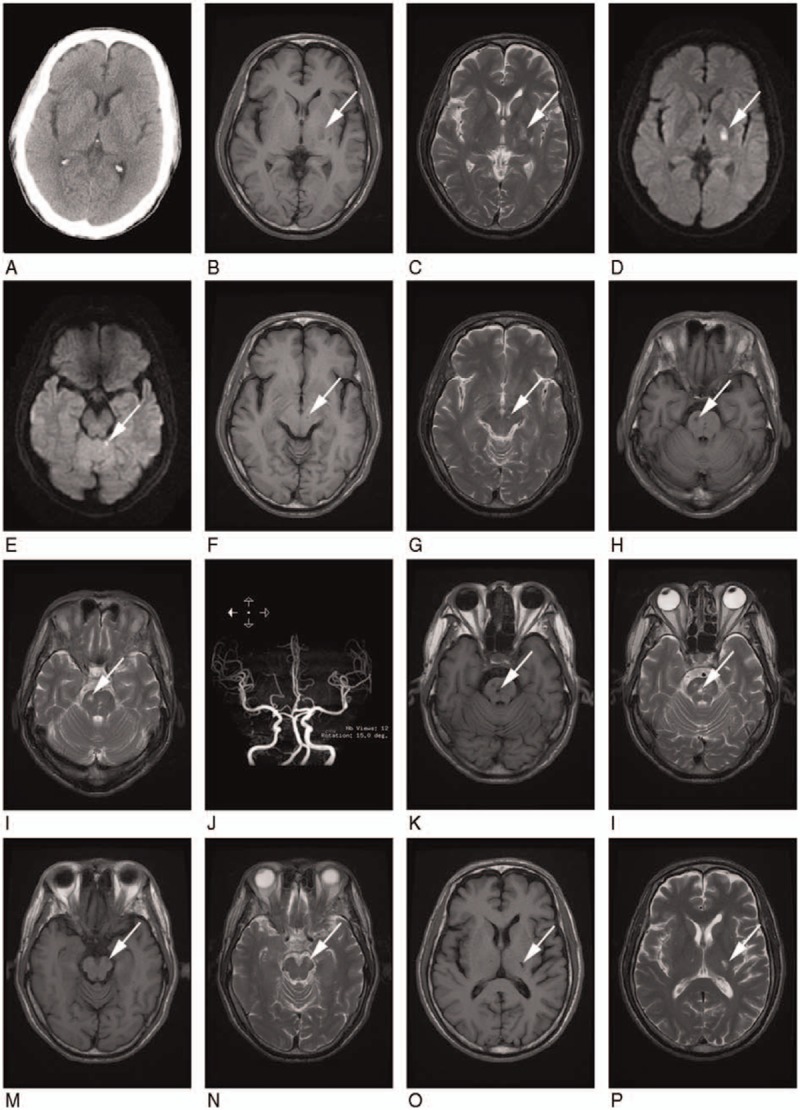
Brain imaging data. At the first admission, a brain CT scan was normal (A), but brain MRI and DWI showed new onset of lacunar infarction involving the left basal ganglia (B: T_1_ WI, C: T_2_ WI, D: DWI), the left vermis (E: DWI); and other lacunar infarction in the left side of the mesencephalon (F: T_1_ WI, G: T_2_ WI), and also the right side of the pons (H: T_1_ WI, I: T_2_ WI). MRA indicated no intracranial artery stenosis, but had stiff blood vessels (J). At the second admission, images revealed multiple lacunar infarcts observed in the pons varolii (K: T_1_ WI, L: T_2_ WI), the left side of the mesencephalon (M: T_1_ WI, N: T_2_ WI), and the right side of the thalamus (O: T_1_ WI, P: T_2_ WI). CT = computed tomography, DWI = diffusion weighted imaging, MRA = magnetic resonance angiography, MRI = magnetic resonance imaging.

A 60-year-old married man, complaining of weakness in his right limbs and difficulty with verbal expression, was admitted to Zhongshan Hospital Xiamen University for the first time on September 4, 2010. He had a history of diabetes mellitus (controlled in normal blood sugar level) for more than 5 years and tobacco use for 30 years. General physical examination was normal except for high blood pressure of 178/115 mm Hg and low-density lipoprotein (LDL) of 3.92 mmol/L (Cholesterol: 5.55 mm mmol/l, Triglyceride:1.355 mm mmol/l). A clinical neurological examination showed that he was suffering from mild expressive aphasia, right central facial palsy, and mild right hemiparesis. Muscle strength in the patient's right limbs was grade 4. In addition, the right Babinski sign was positive. However, sensation was normal, and Kernig sign was negative with a supple neck. The echocardiogram showed no abnormalities with the heart. A brain computed tomography (CT) scan was normal (Fig. 1A), but brain magnetic resonance imaging (MRI) and diffusion weighted imaging (DWI) showed new onset of lacunar infarction involving the left basal ganglia (Fig. 1B–D), the left vermis (Fig. 1E); and other lacunar infarction in the left-side mesencephalon (Fig. 1F and G), and also right-side pons (Fig. 1H and I). Magnetic resonance angiography indicated no intracranial artery stenosis, but had stiff blood vessels (Fig. 1J). A blood specimen taken at the time of admission revealed the patient had negative RPR and specific antitreponemal IgM, and was positive for *T pallidum* particle agglutination (TPPA) (titer, 1:320). Diluted serum was tested to exclude a false-negative test due to the prozone phenomenon of the syphilis RPR. The patient claimed no history of sexually transmitted disease or suspicious clinical symptoms before and around the time of admission, but he admitted to having had extramarital sex 1 time several years ago. Meanwhile, his wife had no history of syphilis or extramarital sex. The patient refused a lumbar puncture examination. The syphilis and neurosyphilis were diagnosed or excluded as described previously.^[7,8]^ The initial diagnoses included: multiple infarction; diabetes; hypertension; and latent syphilis of unknown duration (suggestion: having a lumbar puncture if he experienced continued neurological discomfort to rule out or confirm neurosyphilis). Supporting treatment for cerebral infarction (including management for diabetes and hypertension) was initiated based on the 2008 European Stroke Organization guidelines,^[9]^ and treatment with benzathine penicillin for syphilis (7.2 million units total, administered in 3 doses of 2.4 million units intramuscular injection per dose at 1-week intervals) was according to the US centers for disease control (CDC) guidelines.^[10]^ The patient was discharged with improved function after 30 days.

The patient was re-admitted to the same hospital on April 20, 2011 due to sudden weakness in the right extremities and language disorders. His clinical signs and symptoms were identical as his last visit. A brain MRI and DWI indicated multiple cerebral infarctions observed in the pons varolii (Fig. 1K and L) and the left side of the mesencephalon (Fig. 1M and N), and the right side of the thalamus (Fig. 1O and P). Blood tests revealed negative RPR (ruling out the prozone phenomenon), negative specific antitreponemal IgM, but positive TPPA (titer: 1:640). The patient denied having extramarital sex after the first admission. Two days after admission, lumbar puncture examination indicated that the following: 4.0 × 10^6^/L CSF-WBCs, 413.10 mg/L CSF-protein, a negative CSF-RPR test, and a negative CSF-TPPA test. All tests for microorganisms were negative (Table 1). Clinical laboratory indices for neurosyphilis were all negative. The clinical diagnosis on April 20, 2011 was: multiple cerebral infarct; diabetes; hypertension; and latent syphilis of unknown duration. Supporting treatment for cerebral infarction was only provided according to the 2008 European guidelines,^[9]^ but therapy for syphilis was not provided at this time of re-admission. After 20 days of treatment, the patient was discharged because of increased strength in the right extremities and improved language functions.

**Table 1:**
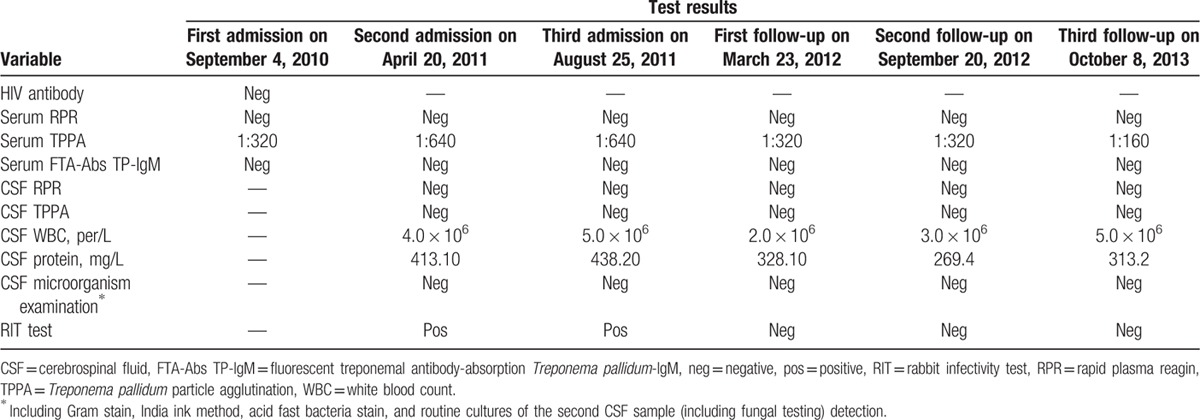
Clinical test data.

However, 1.0 mL of CSF (undiluted) from the patient at his second admission was inoculated into a seronegative New Zealand White male rabbit on April 22, 2011. After 3 months, the rabbit showed neither seroconversion nor orchitis. Then the rabbit was euthanized, and samples from the testes were transferred to a naive rabbit in a “blinded" manner. Twenty-eight days later, the rabbit developed orchitis, and RPR (1:16) and TPPA (1:1280) were positive, indicating seropositivity for *T pallidum*. Furthermore, motile *T pallidum* were observed in isolated testicular fluids under dark-field microscopy. The RIT was therefore positive for *T pallidum*. This syphilis strain was confirmed by DNA subtyping using the form “CDC subtype/tp0548 sequence type,” and the strain type was identified as 14d/f.

On August 25, 2011, the patient was admitted for a third time to the same hospital for re-treatment of syphilis. Before treatment, blood and CSF data were collected (Table 1). Although clinical data did not support a diagnosis of neurosyphilis, the positive RIT by patient's CSF provided definitive evidence that *T pallidum* had invaded the central nervous system (CNS). Treatment with penicillin specifically for neurosyphilis was administered according to US CDC guidelines, as follows^[10]^: aqueous crystalline penicillin G was administered intravenously at 24 million units per day (4 million units every 4 hours) for 14 days. At discharge, the patient was advised to undergo serological testing and CSF examination (including a RIT test for scientific research) every 6 months. No evidence of new cerebral infarct or neuro-invasion was observed during a 2-year follow-up period (Table 1).

## 3 Discussion

European CDC guidelines state that titers of nontreponemal and specific antitreponemal IgM are correlated with disease activity in carriers of active infectious syphilis.^[11]^ However, the European CDC also states that a titer of <1:32 or a negative Venereal Disease Research Laboratory test/RPR test is not sufficient to exclude an active syphilis infection, although the presence of an active treponemal disease in such a patient would be unusual. Although a positive antitreponemal IgM test indicates active infection, a negative antitreponemal IgM test does not exclude active infection, especially if the test is performed during the late stages of infection.^[11]^ Nevertheless, clinicians habitually use negative antitreponemal IgM or Venereal Disease Research Laboratory test/RPR results as the evidence of disease-free and active infection-free status.^[11,12]^ This is the first study showing the successful recovery of motile *T pallidum* using RIT with the CSF derived from a cerebral infarct patient with latent syphilis whose nontreponemal and specific antitreponemal IgM tests were both negative. These results indicate that negative results in nontreponemal and/or specific antitreponemal IgM tests do not exclude the presence of active infectious syphilis.

It is important to select the correct form of penicillin to treat syphilis. This fact reflects the ability of *T pallidum* to sequester in the CNS and/or the aqueous humor, which are sites that are relatively inaccessible to some forms of penicillin.^[13]^ In the present case study, the patient was treated for latent syphilis using benzathine penicillin as a result of his first hospital admission. At his second hospitalization, for cerebral infarct, the clinical symptoms were identical, but a RIT performed using the patient's CSF was positive for motile *T pallidum*, which indicated that the previous treatment for latent syphilis had failed, even though CSF examination did not reveal abnormalities commonly observed in neurosyphilis. The patient was therefore hospitalized and treated with aqueous crystalline penicillin G, which is the commonly recommended treatment for neurosyphilis. Follow-up examinations after intravenous penicillin treatment at 0.5, 1, and 2 years demonstrated that there were no motile *T pallidum* in the CNS after the treatment.

Diagnosing neurosyphilis is a difficult and complex process.^[7]^ After a comprehensive clinical evaluation, the patient in this case study was diagnosed with cerebral infarct, but the clinician did not diagnose him with neurosyphilis. In hindsight, it is not possible to know whether the cerebral infarct and the *T pallidum* infection were associated. Additionally, the *T pallidum* strains identified in the patient's CNS by the tests performed on April 20, 2011 and August 25, 2011 were all types 14d/f, which has previously been considered to be potentially more neuroinvasive and better able to evade immune responses in the CNS.^[14]^

In conclusion, this individual case study shows that negative results in nontreponemal and/or specific antitreponemal IgM tests do not exclude active infectious syphilis. Moreover, selecting the appropriate penicillin preparation is important because *T pallidum* can reside in sequestered sites. This case study also indicates the necessity of treating a patient with known invasion of the CNS with aqueous crystalline penicillin G, if previous treatment for syphilis failed and patients had some clinical neurological presentation that is otherwise unexplained, but that could represent neurosyphilis. One limitation of the current study is that these findings are based on a single case. Therefore, additional studies are needed to confirm the results in other syphilis patients.
